# Expression analysis of DNA methyltransferase and co-repressor genes in *Quercus suber* phellogen: an attempt to correlate with cork quality

**DOI:** 10.1186/1753-6561-5-S7-P169

**Published:** 2011-09-13

**Authors:** Miguel Ramos, Margarida Rocheta, Luísa Carvalho, José Graça, Leonor Morais-Cecilio

**Affiliations:** 1 CBAA, ISA, Technical University Lisbon, Tapada da Ajuda, 1349-017 Lisbon, Portugal; 2 CEF, ISA Technical University Lisbon, Tapada da Ajuda, 1349-017 Lisbon, Portugal

## Background

Cork oak (*Quercus suber*) is one of the most important forest species in Portugal. Cork oak produces a thick cork bark which is harvested for industrial uses. Cork quality is the most important factor that affects its uses technical performance and economic value. Cork quality is associated with many features, the most relevant being the porosity resulting from the phellogen’s differentiation in filling tissue which will degenerate to lenticels. Good cork has few pores of very thin diameter being the opposite valid for low cork quality. Aiming to understand the mechanisms responsible for controlling of the molecular machinery involved in cork production, namely at the epigenetic level, it is relevant to study the expression profiles of the enzymes involved in DNA methylation in the phellogen.

Methylation of cytosines in the DNA, achieved by DNMT methyltransferases, is one of the most important factors in the regulation of gene expression [1]. In plants three DNMT classes have been identified, each one with its function: the CMT (chromomethylase) class found only in plants, is responsible for maintaining the methylation in CpHpG sequences [2]; the DRM (Domain-Rearranged-Methyltransferase) class is associated with *de novo* methylation in any context [3]; and the MET class, responsible for the maintenance of methylation in CpG zones [1]. Proteins, such as the DNA methyltransferase-associated protein (DMAP1), are known to form stable complexes with DNMTs and act as co-repressors of gene expression.

In this work, we report the transcriptional profile of three putative DNA methyltransferase genes from the CMT, DRM and MET classes, and one DMAP in *Q. suber* phellogen of trees producing good or bad quality cork.

## Methods

Three *Quercus suber* potential producers of good quality cork and three producers of bad quality cork were selected. Samples of cork and phellogen were collected from these trees to further estimate cork quality and perform a transcriptional analysis of selected genes. Previously an *in silico* analysis was performed on a cork oak EST library (COEC, Cork Oak ESTs consortium). Three EST sequences, referred as DNA methyltransferases, and one as DMAP1 were selected. Each one was identified and characterized against available databases. Primers were designed to specifically amplify each sequence in real-time-PCR analysis. The actin gene was chosen as internal reference. RNA was extracted from phellogen samples, using Spectrum Plant Total RNA kit and cDNA was obtained with Retroscript kit. Gene expression was analyzed in the six phellogen samples, using qRT-PCR in triplicate reactions performed for each cDNA template with each primer pair. The NormFinder algorithm was used to evaluate the gene expression’s stability using the sample 75 as reference.

## Results

Measurement of cork density allowed forming two groups of trees: (1) three individuals with the lower average density (0.289 g.cm-3 ± 0.037) classified as good cork quality producers, (2) three trees with the highest average density (0.388 g.cm-3 ± 0.036)classified as bad cork quality producers.

Homology search using BLASTP algorithm against available databases showed significant similarities between selected our putatively translated ESTs and known DNMTs (CMT3 - ABW96889, DRM2 - ABW96890 and MET2 - XP_002874265) and DNMT associated proteins (DMAP1- XP_002515237) with e-values of 2e-50, 5e-95, 1e-92, 9e-64, respectively

The expression of the three putative DNMTs and DMAP1 genes was evaluated in the phellogen of both tree groups.

The gene expression stability, evaluated through the NormFinder algorithm, showed values ranging from 0.4 for QsDRM2 to 1.5 for DMAP1. Actin also showed an appropriate value (0.7) to be used as internal control.

**Figure 1:**
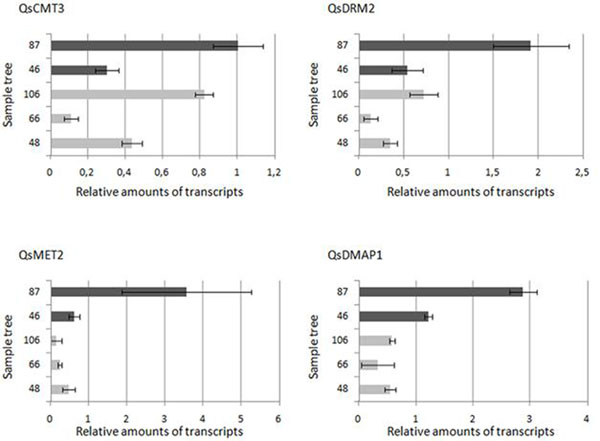
Comparative expression levels of each gene studied. 87 and 46 – bad cork quality producers; 106, 66 and 48, good cork quality producers.

Bad cork quality tree #75 was used as reference. QsMET2 and QsDMAP1 genes showed the highest expression values, while QsCMT3 and QsDRM2 presented the lowest. Comparison of both group trees revealed that, in average, the expression level of each gene is higher in the bad cork quality group than in the good cork quality (Fig1).

## Conclusions

In this work we identified one functional DNA methyltransferase of each class (QsCMT3, QsDRM2 and QsMET2) and the MET1 associated protein (QsDMAP1) in cork oak phellogen. This protein has been described in humans [4] as a co-repressor of gene expression, capable of binding with other regulatory proteins such as histone deacetylases [4] which are also associated with gene silencing.

The general tendency of DNMTs to express less in trees able to produce good cork quality suggests a globally lower methylation level and therefore a lower phellogen gene repression. We can hypothesize that gene silencing is higher in bad quality producers, leading to a higher weight of genes whose expression originates defects.

Gene stability analyses lead us to consider QsDRM2 as the most stable, as was already reported in other species. Therefore, this gene may be a good candidate to be used as reference gene, in future real-time-PCR analyses. This high stability seems to indicate that *de novo* methylation although occurring, has no influence in good or bad cork quality production. Conversely, the more variable expression, and therefore the genes that can potentially affect cork quality are QsMET2 and QsDMAP1 genes.
